# Oral Lipid-Lowering Treatments Beyond Statins: Too Old and Outdated or Still Useful?

**DOI:** 10.1007/s11883-021-00971-y

**Published:** 2021-10-14

**Authors:** Klaus G. Parhofer

**Affiliations:** Medizinische Klinik Und Poliklinik IV, LMU Klinikum, Marchioninistr. 15, 81377 Munich, Germany

**Keywords:** Ezetimibe, Bempedoic acid, Colesevelam, Fibrates

## Abstract

**Purpose of Review:**

For many years, the lipid-lowering armamentarium consisted of statins and/or ezetimibe and/or bile acid sequestrants and/or fibrates. Now, with the availability of new drugs mostly injectables, the field has changed and the role of oral non-statin drugs (including bempedoic acid) must be reevaluated.

**Recent Findings:**

Ezetimibe remains a very important combination partner for statins with continuously increasing treatment numbers. Bempedoic acid is another interesting combination partner for statin/ezetimibe or ezetimibe alone but lacks in contrast to ezetimibe evidence from outcome trials. The role of fibrates is less clear as they have shown disappointing results in outcome trials but may still be used in selected, high-risk patients with combined dyslipidemia. Bile acid sequestrants are now rarely used as there are stronger, better tolerable ways to lower LDL-cholesterol.

**Summary:**

With the introduction of new injectable lipid-lowering drugs, some oral drugs such as ezetimibe and bempedoic acid still have an important spot in our treatment algorithm others such as fibrates have a less clear role while again others are now rarely used.

## Introduction

The knowledge that lipids play an important role in atherogenesis and that lipid lowering translates into risk reduction for cardiovascular events has promoted the development of a number of new lipid-lowering drugs which can dramatically change the level of atherogenic lipoproteins. This is particularly true for PCSK9 antibodies such as alirocumab and evolocumab or PCSK9 inhibitors based on siRNA technology such as inclisiran but is also true for drugs used in rare conditions such as volanesorsen in familial chylomicronemia syndrome. In addition, a number of new drugs addressing ANGPTL-3, apo(a) or apoCIII, or other components important for plasma lipid levels are being developed. Most of these new drugs will be injectables as they are either antibodies or oligonucleotides.

The availability of such potent drugs to modify lipid levels raises the important question of whether and to what extent oral lipid-lowering drugs should be used. Statins are the basic lipid-lowering therapy and will probably remain so for many years as they are cheap, widely available, and supported by numerous convincing outcome trials. In addition, the safety profile of statins is extremely good. However, the positioning of other oral lipid-lowering drugs (Table 1) often used in combination with statins may be less clear. It is questionable whether they can keep their present role considering that the newer injectable drugs mentioned above are usually also used in combination with statins and show a convincing efficacy and safety profile.

**Table 1:** Characteristics of oral lipid-lowering drugs (except statins)

Drug/drug class	Mode of action	Lipid effect	Side effects	Outcome trials (without statins)	Outcome trials with statins
Ezetimibe	Inhibits NPC1L1 → decreased cholesterol absorption → increased uptake from plasma	• LDL-chol: − 20 to − 30% • HDL-chol: No significant effect • TG: No significant effect • Non-HDL-chol: − 15 to − 20%	Rarely myopathy	Not available	Reduction of cardiovascular events (IMPROVE-IT; SHARP)
Bempedoic acid	Reduces cholesterol biosynthesis by inhibiting ACL → increased cholesterol uptake from plasma	• LDL-chol: − 25 to − 35% • HDL-chol: No significant effect • TG: No significant effect • Non-HDL-chol: − 20 to − 30%	Hyperuricemia; gout; elevated liver function tests; decrease in glucose	Not available	Not available
Fibrates	PPAR-α activation → decreased production and increased catabolism of triglyceride-rich lipoproteins	• LDL-chol: − 30 to − 0% • HDL-chol: 0 to − 30% • TG: − 20 to − 70% • Non-HDL-chol: variable	Myopathy; elevated liver function tests	Reduction of cardiovascular events (VA-HIT; Helsinki-Heart-Study)	No effect on cardiovascular events (ACCORD; FIELD)
Bile acid sequestrants	Loss of bile acids → increased production from cholesterol → increased absorption of cholesterol from plasma	• LDL-chol: − 20 to − 30% • HDL-chol: No significant effect • TG: No significant effect • Non-HDL-chol: − 10 to − 20%	Gastrointestinal symptoms; binding of other medications and vitamins	Reduction of cardiovascular events; reduction of mortality (lipid prevention trial)	Not available

The purpose of this review is to pigeonhole the role of these oral drugs in the current environment of new (expensive) injectable lipid-lowering drugs. This review will discuss ezetimibe, bempedoic acid, fibrates, and bile sequestrants.

## Ezetimibe

Ezetimibe is a cholesterol absorption inhibitor, which blocks the Niemann-Pick C1 like 1 protein (NPC1L1) in the gastrointestinal tract (as well as in hepatocytes). Blocking intestinal absorption of cholesterol results in an upregulation of LDL-receptors which consequently reduces plasma LDL-cholesterol levels [1]. Ezetimibe is on the market for almost 20 years.

In combination with statins, ezetimibe can decrease LDL-cholesterol on average by 20–25% although the effect may vary depending on whether the treated subject is a high or low absorber [2]. Ezetimibe is very well tolerated with almost no side effects of its own. Despite this relevant LDL-cholesterol reduction and very benign side-effect profile, ezetimibe was used reluctantly until the publication of the outcome trial in 2015. Since then, the use of ezetimibe alone or in combination with statins has increased and is further increasing. Most guidelines and recommendations now favor statin ezetimibe combination if statin therapy by itself is not sufficient to reach treatment targets [3]. Most recommendations propagate a sequential approach with using statin monotherapy first and then adding ezetimibe if necessary. However, in some patients with severely elevated LDL-cholesterol (large distance to goal), primary combination therapy may be an option. This may especially be the case when follow-up visits are difficult to implement. Ezetimibe is also used in many patients with statin intolerance as ezetimibe itself has basically no side effects [3, 4].

Ezetimibe was tested in a large outcome trial (IMPROVE-IT Study) in which the combination of simvastatin with ezetimibe was compared to simvastatin monotherapy in 18,144 patients with acute coronary syndrome [5•]. The reduction of LDL-cholesterol from 69.5 mg/dl (simvastatin monotherapy) to 53.7 mg/dl (simvastatin/ezetimibe group) resulted in a significant 6.4% risk reduction (0.89–0.99; *p* = 0.016). Further subgroup analysis indicates that particularly patients at high risk benefited from this additional decrease in LDL-cholesterol [6]. The IMPROVE-IT trial can be considered as a milestone study as it was the first study to show that LDL-cholesterol levels around 50 mg/dl are more effective than those around 70 mg/dl with respect to risk reduction. It was also the first study to show that LDL lowering in combination with statins translates into benefit and finally the study gave ultimate proof for the safety of ezetimibe. In a further outcome trial, the combination of simvastatin and ezetimibe was tested in patients with renal insufficiency, again showing significant risk reduction [7]. Although the study lacks a statin monotherapy arm, it is interesting, as previous studies using atorvastatin or rosuvastatin did not show benefit in patients with end-stage renal disease.

Furthermore, newer data indicate that NPC1L1 may also play a role in non-alcoholic fatty liver disease (NAFLD) and non-alcoholic steatohepatitis (NASH) [8]. In fact, a recent meta-analysis shows that ezetimibe can attenuate serum liver enzymes and hepatic steatosis and ballooning in fatty liver disease [9]. However, larger studies including randomized controlled trials are necessary to determine ezetimibe’s clinical role in the management of fatty liver disease.

In conclusion, ezetimibe is an effective and safe drug that can decrease LDL-cholesterol in addition to statin therapy and this additional reduction translates into clinical benefit (Table 2). It can be assumed that ezetimibe will remain the primary partner for statins, if statins alone are not sufficient to reach the LDL-cholesterol goal. Its role in fatty liver disease needs to be determined.

**Table 2:** Use of oral lipid-lowering drugs (except statins) in clinical practice

Drug/drug class	Clinical use	Predicted future role
Ezetimibe	• In combination with statins to further decrease LDL-cholesterol	Use will probably increase
	• Together with low-dose statins (in statin intolerance)	
	• As monotherapy or in combination with other lipid-lowering drugs (in patients with statin intolerance)	
Bempedoic acid	• In combination with low-dose statins and/or ezetimibe (in statin intolerance)	Future role will largely depend on outcome trial
	• In patients not reaching LDL-goal on moderate or high-dose statin plus ezetimibe	
	• In combination with other lipid-lowering drugs (in statin intolerance)	
Fibrates	• In isolated hypertriglyceridemia to reduce risk for pancreatitis	uSe will probably decrease, except if pemafibrate outcome trial is strongly positive
	• In combination with statins (selected patients) to reduce cardiovascular risk in patients with combined dyslipidemia	
	• To decrease LDL-cholesterol, if statins, ezetimibe, bempedoic acid, and/or bile acid sequestrants are not tolerated or not available	
Bile acid sequestrants	• In LDL-hypercholesterolemia if other drugs are not tolerated or not available	Use will probably further decrease
	• As add on to statins and ezetimibe in patients with LDL-hypercholesterolemia not qualifying for PCSK9-inhibition	
	• In LDL-hypercholesterolemia and contraindication to systemic drugs (pregnancy; children)	
	• CAVE: May aggravate hypertriglyceridemia in hypertriglyceridemic subjects	

## Bempedoic Acid

Bempedoic acid is a new oral lipid-lowering drug which has been introduced to the European market in November 2020. Similar to statins, bempedoic acid reduces cholesterol biosynthesis [10]. While statins inhibit HMG-CoA-reductase, bempedoic acid inhibits ATP-citrat-lyase (ACL) [11]. In order to inhibit ACL, bempedoic acid has to be activated by long-chain acyl-CoA-synthetase-1. This activation can only take place in hepatocytes. Therefore, muscle-related side effects seen with statin therapy in approximately 10% of patients should not occur when patients are treated with bempedoic acid. This is supported by data from phase 2 and 3 studies, in which it was shown that muscle-related side effects are at placebo level [12••].

Although bempedoic acid inhibits the same pathway as statins, bempedoic acid can decrease LDL-cholesterol even in patients treated with statins [12••, 13–15]. However, the effect on LDL-cholesterol is less pronounced when used with high-dose statins. In monotherapy, LDL-cholesterol can be decreased by approximately 20%. In combination with ezetimibe, reductions of up to 35% can be observed.

Bempedoic acid may increase the dose of statins; therefore, the combination with high-dose simvastatin should be avoided [16]. Furthermore, bempedoic acid can increase uric acids levels which in predisposed patients may lead to hyperuricemia or gout. In phase 2 and phase 3 studies, increases in uric acid levels are observed in approximately 2% of patients, while acute gout occurred in 1.4% (compared to 0.4% on placebo). The risk of gout is considerably higher in patients with a history of gout (11% vs. 2.9% on placebo). Other rare adverse events relate to a small decrease in hemoglobin levels, the mechanism behind this is unclear. Finally, the use of bempedoic acid is associated with decrease in glucose levels (in contrast to statins) and also significantly decreases CRP (similar to statins).

Currently, no outcome data are available but it is expected that the CLEAR outcome study which examines the effect of bempedoic acid compared to placebo in statin-intolerant patients (> 14,000 patients, approximately 4.75 years observation period) will be presented in 2023. Genetic data strongly indicate that LDL-cholesterol reduction achieved with bempedoic acid will result in risk reduction [17].

Currently, bempedoic acid is mostly used in 2 clinical settings [16]. Primarily in patients with statin intolerance in whom it can be assumed that a decrease LDL-cholesterol by approximately 30–40% will be sufficient to reach the treatment target. In that situation, bempedoic acid can be used together with different doses of statins and ezetimibe. Furthermore, bempedoic acid may also be used in situations where patients at high or very high risk do not reach the treatment target despite high-dose statin therapy in combination with ezetimibe. However, this approach is usually only successful if the distance to treatment target is not “too far.”

In summary, bempedoic acid is an interesting new lipid-lowering drug, which is primarily used in statin-intolerant subjects but may also play a role in patients not reaching LDL-cholesterol target despite maximal statin end/or ezetimibe and/or PCSK9-based therapy (Table 2). The future positioning of bempedoic acid will largely be determined by the results of the outcome trial.

## Fibrates

Fibrates bind and activate PPAR-α [18]. Although the exact mechanism is not clear, the fibrate-induced activation of PPAR-α results in a decrease in apo CIII, an activation of lipoprotein lipase, and apo AV but also decreases the activity of diacylglycerolacyltransferase [19]. At the same time, apo AI and apo AII levels are increased. Overall, this results in a significant decrease of triglyceride levels (up to minus 70%) and an increase in HDL-cholesterol (up to plus 30%). The effect on LDL-cholesterol is variable and probably depends on the underlying dyslipidemia. While the primary action of fibrates seems to be the reduction of triglyceride levels, it is unclear whether the changes observed in HDL-metabolism are the result of PPAR-α activation or are just secondary to the triglyceride reduction.

In contrast to statin therapy, the effect of fibrate therapy is less predictable. This most likely represents the fact that there is a wide range of potential causes for hypertriglyceridemia (usually a combination of genetic predisposition and lifestyle factors). It should be noted that triglyceride reduction with fibrates is minimal in the most severe cases of hypertriglyceridemia such as familial chylomicronemia syndrome [20].

In monotherapy fibrates (usually gemfibrocil) can reduce the risk for atherosclerotic disease, but in combination with statins (used fibrate fenofibrate), no additional benefit was observed [21–24]. However, subgroup analyses of these outcome trials indicate that such combination therapy may reduce the risk in patients with hypertriglyceridemia and low HDL-cholesterol [25•]. It is currently unclear whether the failure to show risk reduction in combination trials relates to true failure or to methodological aspects related to study design. Pemafibrate, a new potent fibrate, is currently being tested in a large outcome trial and results will probably be reported in 2021 or 2022 [26, 27].

While the currently available outcome trials are disappointing with respect to cardiovascular risk reduction, they surprisingly showed a significant effect of fenofibrate on microvascular complications in diabetic patients [28]. The effect was particularly strong for retinopathy in the FIELD trial and resulted in the approval of fenofibrate for treatment of diabetic retinopathy in some countries.

From a clinical perspective, fibrates are used in patients with hypertriglyceridemia either as monotherapy in patients with isolated hypertriglyceridemia and low cardiovascular risk or in combination with LDL-cholesterol-lowering drugs if LDL-cholesterol is reached and risk is perceived as high or very high. No clear-cut recommendations when to use fibrates are currently available, which is mostly due to the lack of convincing outcome trials (as outlined above) [3, 29].

In conclusion, fibrates still play a role in patients with combined dyslipidemia or isolated hypertriglyceridemia but due to the lack of convincing outcome trials, the decision should be individualized (Table 2). In addition, it should be noted that soon, eicosapentainic acid ethylester will be available which may further decrease the role of fibrates (especially if the pemafibrate trial is negative).

## Bile Acid Sequestrants

Bile acid sequestrants such as colesevelam, colestipol, or colestyramine bind bile acids in the gut and thereby prevent the reabsorption of bile acids. This results in the loss of bile acids from the enterohepatic circulation. This depletion of bile acids leads to an increased production of bile acids for which cholesterol is required [30, 31]. Therefore, hepatic uptake of cholesterol from the circulation is increased (as well as biosynthesis). A daily dose of 8–24 g of cholestyramine and 3.75 g of colesevelam must be used to induce significant decreases of LDL-cholesterol. Interestingly, colesevelam has consistently shown glucose-lowering effects, which is particularly noteworthy as statins increase glucose levels [32]. Despite this, its role in diabetes treatment needs to be determined.

Typically, reductions in LDL-cholesterol of 20–30% can be observed. Importantly, triglyceride levels may increase during bile acid sequestrants therapy because the production of bile acids and VLDL is co-regulated [33]. Despite this significant effect on LDL-cholesterol, these drugs are rarely used in current practice because of the high rate of side effects. These include gastrointestinal symptoms, most commonly flatulence, constipation, dyspepsia, and nausea. Furthermore, the absorption of fat-soluble vitamins can be reduced. As bile acid sequestrants can also bind to many commonly prescribed drugs, they must be administered either 4 h before or 1 h after other drugs. Compared to colestyramine and colestipol, colesevelam is better tolerated and has fewer interactions with other medications. On the other hand, bile acid sequestrants are not systemically absorbed and can therefore be used in children and pregnant women.

Bile acid sequestrants have shown benefit in large outcome trials (before the availability of modern treatment options) [34]. No outcome data are available in combination with statin therapy.

Currently, bile acid sequestrants are only used as third-line options for decreasing LDL-cholesterol and are only used in selected patients and situations (Table 2). It can be assumed that with newer and more potent drugs available to decrease LDL-cholesterol, the role will be further reduced.

## Conclusion

Although a number of very potent, very effective, and safe lipid-lowering injectables are being developed or introduced to the market, there remains space for oral lipid-lowering drugs besides statins. Some of them, such as ezetimibe and bempedoic acid, will probably further grow, while the future of others, such as fibrates and bile acid sequestrants, is less clear.
